# The expression of ultimate life goals in co-creative art processes with palliative cancer patients

**DOI:** 10.1186/s12904-023-01294-2

**Published:** 2023-11-02

**Authors:** Yvonne Weeseman, Michael Scherer-Rath, Nirav Christophe, Henny Dörr, Esther Helmich, Mirjam A. G. Sprangers, Niels van Poecke, Hanneke W. M. van Laarhoven

**Affiliations:** 1 Department of Medical Oncology, Amsterdam University Medical Centers, University of Amsterdam, De Boelelaan 1117, Amsterdam, 1081 HV The Netherlands; 2 Cancer Center Amsterdam, Treatment and Quality of Life, Amsterdam, The Netherlands; 3 Faculty of Philosophy, Theology and Religious Studies, Radboud University, Nijmegen, The Netherlands; 4 HKU University of the Arts Utrecht, Utrecht, The Netherlands; 5 Amsta Healthcare Organization, Amsterdam, The Netherlands; 6 Amsterdam UMC location, Medical Psychology, University of Amsterdam, Amsterdam, The Netherlands; 7 Amsterdam Public Health, Mental Health, Amsterdam, The Netherlands

**Keywords:** Advanced cancer patients, Art, Co-creation, Experiences of contingency, Integration of experiences of contingency, Life narratives, Quality of life, Ultimate life goals

## Abstract

**Background:**

Co-creation, characterized by artists and patients creating a joint work of art, may support patients with the integration of disruptive life events into their life story, such as living with cancer. Focusing on experiences of contingency and life goals could support this process. The research questions are: (1) ‘how are patient’s ultimate life goals and experiences of contingency expressed in the work of art as created in a process of co-creation?’; (2) ‘how do the four phases of integration of experiences of contingency unfold during co-creation?’

**Methods:**

Ten patients who were in a palliative stage of cancer treatment completed co-creation processes. Audio recordings of these co-creation processes were imported in Atlas-Ti and analysed by applying directed content analysis. We searched for life goals and experiences of contingency in the four phases of co-creation; *Art communications, Element compilation, Consolidation, Reflection.*

**Results:**

Patients used 4–8 sessions (median 5 sessions) with a duration of 90–240 min each (median duration 120 min). All patients expressed their experience of contingency and their ultimate life goals within the four phases of co-creation and in their work of art. A case description is presented illustrating the co-creation process.

**Conclusions:**

During co-creation, patients move through four phases in which experiences of contingency and ultimate life goals can be made explicit through art making and can be expressed in the work of art, supporting integration of experiences of contingency into one’s life narrative.

## Background

A life narrative can be described as an internal representation of one’s experience of oneself and the world. Life narratives are expected to provide a sense of coherence and meaning, supporting people to successfully navigate the world [1]. Our worldview, and our most prominent aims and goals in life, our ultimate life goals, shape the way we fundamentally perceive the world, form the foundations of our life narrative and influence our actions in life [2, 3]. Our worldview and ultimate life goals can be threatened by life events, such as the diagnosis of cancer, which can disrupt our life narrative [2, 3]. These types of life events are of a contingent nature, as Luhman describes these are “neither impossible nor necessary, it could have happened otherwise” [4]. Such contingent life events can irreversibly introduce a new and undesired future in which one’s existence and life goals are threatened [3, 5]. This can be perceived as an existential crisis when the devastating disruption of one’s life narrative makes one wonder and even doubt one’s own life’s fundamentals [6–9]. An existential crisis evokes emotions like despair, loss, grief and powerlessness [10].

People’s ultimate life goals have been described as a person’s strivings and meaningful objectives, which are consciously pursued in daily life [11, 12]. Ultimate life goals give expression to one’s concerns regarding meaning, existence, purpose and value in combination with one’s worldview [13]. Ultimate life goals form one’s deepest motivations in life and when an event is appraised as relevant to these ultimate life goals, an emotional response is triggered [13]. The importance of ultimate life goals is related to meaning and is eminent in its association with life satisfaction and happiness and its reversed association with depression and disengagement [11]. In general, four main themes of ultimate life goals can be distinguished; work/achievement, intimacy/relationships, spirituality/religion, and, self-transcendence/generativity [12].

Integrating theory on life narratives by Ricoeur [1] and contingency [4, 5, 14], our group [2] has developed a theory on the presumed working mechanism of how people deal with such disruptions of their life narrative. Kruizinga and Hartog hypothesized that, in order to restore the coherence of the life narrative, the experience of contingency needs to be integrated into one’s life narrative during a process which was named narrative integration [2, 6, 9].

As part of a search for potential viable interventions facilitating integration of experiences of contingency into one’s life narrative [15], we are currently investigating a process of co-creation between artists and patients with incurable cancer [16]. Such a co-creation process involves multiple sessions in which a professional artist works together with a patient creating a work of art, while reflecting upon aspects of the patient’s life narrative, including experiences of contingency [16–21]. In a previous investigation on co-creation, based on artists’ retrospective reports of co-creations with a wide variety of patients, building forth on Ricoeur [1], we recently developed a theory on the integration of experiences of contingency within a co-creation setting and found that, during co-creation, patients went through a process of integration of experiences of contingency with a sequence of four phases: art communications, element compilation, consolidation, and reflection [16]. The previous investigation showed the created work of art to reflect patients’ experiences of contingency [16]. However, to the best of our knowledge, the reflection of experiences of contingency in the work of art, and the integration of experiences of contingency in four phases, has not yet been empirically studied in palliative cancer patients. Also, the presence of life goals in the process of integration of experiences of contingency has not yet been explored during integration of experiences of contingency within a co-creation setting.

To deepen our understanding of the process of integration of experiences of contingency, our research questions are: (1) ‘how are patient’s ultimate life goals and experiences of contingency expressed in the work of art as created in a process of co-creation?’; (2) ‘how do the four phases of integration of experiences of contingency unfold during co-creation?’

## Methods

### Participants and intervention

The current study builds on previous research [15, 16, 21] in the *In Search Of Stories *project (ISOS) by investigating completed, audio-recorded co-creation processes in which palliative cancer patients reflected on experiences of contingency. In ISOS, palliative cancer patients participate in a narrative multimodal intervention aimed at enhancing quality of life [15]. Professional artists and spiritual counsellors support participating patients in reading selected literature [15], a life review interview, drawing of Rich Pictures [22] and a co-creation process [16]. The co-creation processes were expected to entail up to 4 sessions of approximately 1 h each. Co-creation is an interactive process with input from both patient and artist where product and process are equally important. Within ISOS, the patient, together with a professional artist, creates a work of art while reflecting upon aspects of the life narrative. Patients are invited to share ideas and, if possible, to practically participate in the construction of the work of art. Practical participation is not mandatory, as for instance a lack of skills or health conditions could be a hindrance, The practical construction of the work of art is the responsibility of the artist. These conditions are expected to give sufficient space for a natural unfolding of creative processes where patients focus on experiences of contingency and co-create a work of art [16, 21]. In total, 25 patients were involved in the ISOS project, of which 10 had finalized the co-creation process at the time of analyses for the current study. Of the other 15 patients, 11 had not yet finalised their co-creation process and four patients had not been able to finalise their co-creation process due to deteriorating health. Inclusion criteria for the patients in the ISOS project were: (i) being in the palliative phase of cancer treatment, (ii) having a diagnosis of metastasized cancer less than three years prior to enrolment, (iii) having sufficient mental, (iv) verbal and physical capacity to participate, and (v) having a life expectancy beyond the planned duration of the ISOS intervention. Inclusion criteria for the current study were: (i) participating in the ISOS project and (ii) having finalised the co-creation process. The 11 professional artists participating in ISOS were selected through the University of Arts Utrecht and had previous experience with co-creation with patients. Inclusion criteria for the professional artists in the ISOS project were: (i) having extensive experience in co-creation processes, (ii) being able to work with multiple art modalities.

### Study design and data collection

The current study followed a qualitative study design in which seven professional artists audio recorded all sessions of co-creation with ten palliative cancer patients within the ISOS project. Artists sent the audio recordings to the researchers for further analyses. In addition to the audio recordings, photos of the co-creative processes were collected.

### Data analyses

The audio recordings of the co-creation sessions were imported in AtlasTi [23] and primarily analysed by YW (MA, MSc, MSc, female), who has a professional background in art therapy, clinical psychology and spiritual care. We have used directed content analysis to investigate our research questions [24]. Directed content analysis is a deductive approach specifically suitable to search for the presence of already established concepts, called ‘category definitions’, and to validate or conceptually extend an already existing theoretical framework. Category definitions regarding the first research question (expression of ultimate life goals and experiences of contingency in the work of art) were based on theoretical work by Hartog et al. (2020): [2] *(1) Experience of contingency*, and, *(2) Ultimate life goals*, and one category definition was theoretically building further on Weeseman et al. (2022): [16] *Expression in the work of art*. The category definitions regarding the second research question (unfolding of the four phases of integration of experiences of contingency during co-creation) were based on Weeseman et al. (2022): [16] *(1) Art communications, 1.1 Tracing different aspects within the life story of the patient, 1.2. Communication through the senses and choice of art material, (2) Element compilation, (3) Consolidation, (4) Reflection*. See Table 1 below for an overview of the category definitions. Category definitions and all data analyses on a general group level and individual level, were extensively discussed during sessions with MSR (Associate professor, PhD, male), who has a professional background in religious studies, theology, spiritual care and qualitative research, and HvL (Professor, MD, PhD, PhD, female), who has a professional background in medical oncology and theology, until consensus was reached. HvL, MSR and YW share a background in theology and spiritual care and additionally, the extensive practical and theoretical background with palliative cancer patients of HvL, the experience with recognition and working with contingency and existential crisis of MSR and the ability to analyse artistic expressions combined with psychological meanings of YW, contributed to the data analysis. The original language of the audio-recordings was Dutch. Excerpts presented here are translated into English by YW.

**Table 1:** Category definitions for the concepts used in the research questions

Research question	Category definition	Description
How are patient’s ultimate life goals and experiences of contingency expressed in the work of art as created in a process of co-creation?	1. Experience of contingency^a^	The experience of a disruption of one’s life narrative, possibly to the extent of an existential interpretation crisis, caused by a conflict between a life event and one’s ultimate life goals and world view. The life event, which was “neither impossible nor necessary, it could have happened otherwise”, irreversibly introduces a new and undesired future, in which one’s existence and life goals are threatened. This makes one wonder and even doubt one’s own life’s fundamentals.
	2. Ultimate lifegoals^a^	People’s personal goals come forth from their beliefs and world view. One’s goals are anchored and justified by one’s world view, which determines the meaning one attributes to a life event. Ultimate meaning in one’s life comes from one’s ultimate life goals or values. Ultimate life goals and values are formulated in an abstract way and cannot be replaced by something else.
	3. Expression in the work of art^b^	The expression in the work of art of one’s experience of contingency in combination with an evaluation of one’s ultimate life goals.
How do the four phases of integration of experiences of contingency unfold during co-creation?	1. Art communications^b^ 1.1 Tracing different aspects within the life story of the patient 1.2. Communication through the senses and choice of art material	The patient’s life story is explored by looking for important elements that are valuable to the patient. These so-called ‘different voices’ within the life story can sometimes oppose each other. Aspects of the life story are uncovered which the patient was not (fully) aware of. Experiences of contingency within one’s life story are explored by using all the ‘senses’—touch, smell, hearing, vision, bodily sensations -- and emotions as a first entry. A further elaboration of these experiences of contingency unfolds by using one specific aspect of the senses to focus on certain elements in more detail. Subsequently, specific art materials are linked to the exploration of these experiences of contingency and used for a further exploration and expression of inner feelings.
	2. Element compilation^b^	Various combinations of the elements originating from the expression of the ‘different voices’(see 1. Art communications) are put together, representing alternative story lines of the life narrative. The iterative combinations of these elements lead to compilations which create a new perspective on one’s life story
	3. Consolidation^b^	The artist together with the patient or the artist alone create the actual work of art. The elements that came forth from the patient’s expression inspire the design of the art work.
	4. Reflection^b^	Patients can simultaneously reflect upon the created work of art, their new life narrative and on the (co-creation) process of arriving both at the work of art and at their new life narrative. Standing on its own, the work of art has a capacity to transcend time. The patient can allocate new meaning to the work of art in his/her own unique way every time he/she observes the work of art.

^a^Category definition described after Hartog et al. (2020) [2]

^b^Category definition described after Weeseman et al. (2022) [16]

### Ethics

As the Medical Research Involving Human Subjects Act was not applicable (reference number: W20_436 # 20.483) the study was exempted from ethical approval by the Medical Ethics Review Committee of the Academic Medical Centre. At the start of their enrolment written informed consent was obtained from each participating patient and artist.

## Results

### Patients, artists and process characteristics

Patients and artists were paired based on patients preference for specific art modalities. Both patient and artist could suggest per session where the co-creation session would take place, on which the patient decided. Table 2 shows the characteristics of the patients and the artists with whom the patients were partnered during the co-creation process. The analysed co-creation processes took place between March 2021 and August 2022, at the professional artist’s studio, at the home of the patient or occasionally at another location, for example, a museum or a forest. Due to differences between patients in the development of creative ideas, the expression of these ideas in one or more initial artforms and the construction of the work of art itself, the co-creation processes entailed four to eight sessions. The median number of sessions was five (range 4–8). The median duration of the co-creation sessions was 120 min (range 90–240 min).

**Table 2:** Participating patients, artists and patient-artist dyads

Patient	Age	Gender	Worldview	Cancer diagnosis	Artist	Age	Gender
1	45	Female	Catholic	Cervical cancer	Visual artist (A)	38	Female
2	71	Female	Multiple religious belonging^a^, elements of Buddhism	Cholangio cancer	Scenographer (B)	50	Female
3	57	Female	Multiple religious belonging	Bile duct cancer	Visual artist (A)	38	Female
4	59	Female	Multiple religious belonging, elements of Buddhism	Colon cancer	Musician (C)	43	Male
5	66	Male	Multiple religious belonging	Oesophagus cancer	Musician (D)	45	Male
6	81	Male	Catholic	Oesophagus cancer	Musician (D)	45	Male
7	55	Female	Multiple religious belonging, native American spirituality	Pancreatic cancer	Scenographer (E)	50	Female
8	61	Female	Catholic but not actively worshipping	Breast cancer	Visual artist (F)	64	Male
9	68	Female	Multiple religious belonging, catholic upbringing	Rectal cancer	Visual artist (G)	52	Female
10	64	Female	Multiple religious belonging, catholic upbringing	Neck cancer	Scenographer (B)	50	Female

^a^Multiple religious belonging is a broad term describing one’s beliefs originate from multiple religions [25]

### Research question 1: experiences of contingency, ultimate life goals and the work of art

#### Overview of the expression of experience of contingency and ultimate life goals in the work of art

For seven out of ten patients, the main experience(s) of contingency they reflected upon during the co-creation process, and which were expressed in the work of art, were related to living with cancer in the palliative phase, for example experiencing fear of physical and/or emotional pain related to being ill, feeling alone during the illness, or feeling fear about the uncertainty of the illness trajectory. For three other patients the main experiences of contingency were related to the loss of a partner, experiencing difficulty in expressing multiple facets of oneself, and experiencing loneliness. In addition, some patients also reflected upon and expressed a lack of being valued and/or receiving love as their main experience(s) of contingency, and were actively attempting to deepen connections with significant others during the co-creation process.

The main themes in patient’s ultimate life goals revolved around sharing and receiving (God’s) love, connection to others, experiencing self-worth and connecting or surrendering to an inner or ultimate state of stillness and acceptance. Other ultimate life goals included self-expression, experiencing trust in life’s ways and freedom in living life to its fullest while refraining from emotional suffering. Patients were actively orienting their actions to be in line with these ultimate life goals during the process of being ill.

For all patients the work of art came forth from a combination of their personal preferences of art modalities, the input from the artist, the experiences of contingency they reflected upon, and the ultimate life goals that were present during co-creation. In Table 3 the experiences of contingency, ultimate life goals and works of art are presented for all patients.

**Table 3:** Experiences of contingency, ultimate life goals and the work of art

Patient	Experience of contingency	Ultimate life goals	Works of art	Illustration of the work of art
**1**	Experiencing loneliness during the illness trajectory	Experience (God’s) love and sharing (God’s) love	A blanket with a projected uterus consisting of flowers, representing a loving and nourishing environment. A picture of the patient sleeping under the blanket. A poem the patient had written	
**2**	After several years of being palliative, feeling fear about the uncertainty of the illness trajectory	Freedom in living life to its fullest and refrain from emotional suffering	Several art works consisting of used bandages from chemotherapy treatment, representing lifecycles and ending this period in life	
**3**	Difficulty in accepting being palliative and fear of emotional and physical pain	Experiencing trust in life’s ways and experiencing connection to others	An art work consisting of leaves that represent a phoenix arising from the ashes of previous fears, and being supported by loved ones	
**4**	Fear of physical pain during the illness and the process of dying	Surrendering to a state of inner stillness, a state where acceptance is present	A designed hiking map meant to experience moments of stillness and reflection, representing the road to find an ultimate stillness as a state of being	
**5**	Difficulty accepting that time is getting shorter to experience connection with loved ones	Connecting to loved ones	A musical movie representing life’s journey leading to the light with support from significant loved ones	
**6**	The death of a life partner and subsequently experiencing meaninglessness and lack of connection in life	Experiencing connection to others	A musical movie containing seraphine music performed by the patient in the church that recently became his new place of connection and meaning	
**7**	Regret about missing the warning signal to start pursuing ultimate life goals earlier in life	Becoming one with the ultimate stillness, experiencing acceptance and a wider view on life’s struggles	A wool felt garment embroidered with feathers, representing an attitude of softness and finding a wider perspective in welcoming life as it is	
**8**	Difficulties expressing multiple facets of herself	Self-expression in work and family	A full body portrait with passionate bright colours representing experimenting with intense expression	
**9**	Experiencing loneliness in connection to others	Experiencing self-worth and receiving and sharing love in connection to others	A self-portrait consisting of multiple drawings containing elements of her life, representing all that is important to connect to in life	
**10**	Experiencing fear for the moment of dying and entering the afterlife	Experiencing self-worth and receiving and sharing love in connection to others	A paper-mache bridge constructed of paper from shredded diaries, representing her spiritual transition from earthly life to the after life, guided by the love and connection of significant others	

#### Patient’s quotations of the expression of experience of contingency and ultimate life goals in the work of art

For four patients, illustrating a distinct variety in experiences of contingency and ultimate life goals, the expression of these are presented in more depth and illustrated with quotations based on excerpts from the recordings of the co-creation sessions.

Patient 4 experienced fear of physical pain during the illness and in the dying process. Her ultimate life goal was to surrender to a state of inner stillness, a state where acceptance is present. She designed a hiking map meant to experience moments of stillness and reflection, representing the route to find ultimate stillness as a state of being. As the patient explained: *“Humans have for years been inspired, they have had kids, they have lived and done so much. I do not want to express bitterness as I do not feel bitterness. I want to express the wonders of nature, everyday being a miracle, like for example poppy flowers opening up. I want to emphasize the beauty of nature, and not the fear surrounding cancer. Everything I have done was with a sense of decorum, at a party we dress nice, and in life we dress nice. Otherwise, it is so very boring. It would be shallow if life made no sense at all. Buddhism attracts me because I love the decorum of it. So, it’s going to be a silent walk that differs from other silent walks because it is a walk into ultimate stillness.”*

Patient 5 experienced difficulty in accepting that, due to the illness, time is getting shorter to experience connection with loved ones. His ultimate life goal was connecting to loved ones. He made a musical road movie representing life’s journey leading to the light, with support from significant loved ones. In the words of the patient: *“This black part next to the road, the cancer, is present, but we are slowly moving the other way. The mountains symbolise the road to hope. When the end gets nearer, and the road is bending to the right, and this beautiful sunset is appearing, this represents hope, that things are going to get lighter. I would really like it if the lighting at the end of the movie is going to be much brighter, representing the openness I now experience with my loved ones. I also would like to add all the notes I wrote to all the people that reached out to me.”*

Patient 7 experienced regret that due to a hectic life before falling ill, she missed the warning signal to start pursuing her ultimate life goals earlier in life. She created a wool felt garment embroidered with feathers, representing an attitude of softness and finding a wider perspective in welcoming life as it is. As the patient explained: *“In this work of art, memories and other things that existed unconsciously under the surface are finding their place now. While at the beginning I was reading the story of Orpheus and Euridice, it became so clear to me that both of them are inside of me. You could literally say that he had to rescue her from the underworld, but taking a more abstract perspective, one could also see that he gave her the possibility to get closer to herself. It is just bizarre how we run past life. It took so long for me to find out what I wanted in life. I am really trying to reach for the essence of life now, and making this embroidery with feathers allowed me to find the stillness I was craving for. Bitterness is really not the right word when this illness befalls you. It is that coercion to take a step further in life, and I am so glad that I did this now.”*

Patient 10 experienced fear for the moment of dying as, due to the severity of her illness, the moment of death came nearer. This patient created a bridge constructed of papers from shredded diaries, representing her spiritual transition from earthly life to the afterlife, guided by the love and connection of significant others. In the words of the patient: *“I feel fortunate to be allowed to prepare the people around me, touching others and being touched is the most powerful thing to do right now. They can bring me to the bridge and help me to cross it. And then letting me go, because letting go is the hardest thing. To be able to enter the other world one has to let go, that is essential in this process. I have been wondering a lot how I can bring people to an inner place to let me go when the time is near. The people who will sit around my bed have to know that I need to be taken to this bridge. The narrower part of the bridge represents the most scary part of it, the end is more broad, which represents the easy part. When I have crossed the bridge, all will fall into place by itself.”*

### Research question 2: phases of integration of experiences of contingency

The data necessitated two minor changes in the category definitions used to investigate how the four phases of integration of experiences of contingency unfold during co-creation (see Table 2). The order of sub theme 1.1 and 1.2 was reversed as patients rather tended to start with an exploration of the senses than with an exploration of the life narrative. Phase 2. *Element compilation* was further characterised by two sub themes which are described below. See Table 4 for an overview.

**Table 4:** Phases of integration of experiences of contingency within a co-creation process

Patient	1. Art communications		2. Element compilation		3. Consolidation	4. Reflection
	1.1 Art communications – exploring through the senses	1.2 Art communications - tracing different aspects	2.1 Element compilation – broad arrangement	2.2 Element compilation – acuminating		
**1**	1. Reading the story ‘Farewell from Phoebe’^a^ 2. Images 3. Withered flowers 4. Roses 5. Fabrics	1. Reflecting upon her inability to have children 2. Little girl with her parents 3. Transient nature of her body 4. God’s guidance 5. A dress that her mother once made	Making and subsequently photographing the flower collage which was arranged in the shape of a uterus	Looking for the most fitting arrangement of the projected photoprint to fit a blanket	1. The blanket 2. Photographing her as she was sleeping under the blanket 3. A poem she had written	“This blanket is a warm wrapping of love, a safe consisting of my feelings. I am able to say: let go and let God” (i.e. ’I surrender to God’s plan’)
**2**	1. Used bandages 2. Wool fabrics 3. Stones and shells 4. A piece of the Djoser pyramid	1. Bandages were collected during the chemotherapy treatment 2. She always loved knitting 3. Collecting pieces of the earth she once inhabited 4. Working in Egypt	Knitting a turquoise scarf Using a 3D pen to create a coral necklace	Adding stones and shells to knitted wool and creating a nautilus shape with dyed ribbons of bandage.	Connecting the remaining coloured bandage while making a necklace	“This work shows a sense of gratefulness in the additional time that was granted to me. The chosen colours represent the aura of a person leaving this world.”
**3**	1. Creating curved shapes using clay 2. Drawing of the body consisting of a cut out liver 3 .The colours yellow and red	1. “This is a protective female snake.” 2. “The liver represents my anger and fear and I don’t want that.” 3.”Red and yellow represent passion.”	Arranging 5 curved leaves, where the centre represents graceful femininity. And the other leaves represent protection and connection	Strengthening the leaves by pins, and using hydrochloric acid to create yellow-red corrosion	Finding the right supporting base for the leaves to stand on	“The work expresses the pain of the illness experience, I feel like a phoenix that arises from that pain. The connection and protection from my loved ones is represented in the leaves around me.”
**4**	1. Reading and rewriting the story of Orpheus^a^	1. Identification with Euridice and Orpheus as her husband, both trying to negotiate with Hades to prevent her from dying before she is ready	Going on a hike in nature to find elements representing the natural course of life and death	Creating a hiking map and formulating instructive sentences to accompany the map	Dividing the map in three parts, accompanied by distinct piano and chime music, leading the hiker to a deeper experience of stillness	“The presence of the stillness in nature helps me to see the mortality in nature as a known fact representing my own mortality.”
**5**	1. Drawing a Rich Picture of a road with heavy storm, a burning bush, and light at the end of the road 2. Experimenting with electrical guitar and sounds of thunder	1. “The heavy storm is the cancer on my path in life.” 2. “The guitar music is melancholic suffering, but it is not allowed to take over.”	Creating a film showing a scenic motorbike route accompanied by melancholic guitar music	Letters of thanks to friends are being added, as a symbol of gratitude for their support	The film of his life’s journey is being placed on a website. The music is being printed on a long playing vinyl record and the drawing is used as artwork on the album cover	“The motorbike is passing the cancer on the road, and with the support of others the journey is continued to its end. There is bright light at the horizon.”
**6**	1. Talking about multiple technical elements of the construction of a seraphine	1. Creating and playing music on seraphines has been a vital element throughout his life and connects to many memories	Compiling several romantic pieces of seraphine music and explaining how he feels while listening and playing those musical pieces	Filming in the church where he plays the seraphine almost daily	A film of his hands as he plays the seraphine, accompanied by delayed seraphine music. A photo still of his hands as he plays seraphine	“This film and photo are an impression of me. They reflect playing in the church and also the moments that I feel melancholic about living without my wife.”
**7**	1. Reading the story of Orpheusª and listening to music inspired by the story 2. The colour white while painting an egg 3. Sensing bird feathers	1. “I am going to try to retrieve Euridice: she represents my tenderness that has been neglected for years.” 2. The year’s cycle 3. The inner tenderness-being able to transcend above everything worldly	Felting a woollen garment in the shape of an egg	Embroidering feathers and braided horsehair into the garment	Selecting pictures reflecting the creation process of the garment and making a booklet	“The wool felt garment represents an attitude of tenderness and of finding a wider perspective in welcoming life as it is. Understanding why things had to happen in a certain way.”
**8**	1. Reflecting upon portraits during a Renaissance exposition 2. Exploring the use of colours in pictures of nature	1. “I am not really aware of status sensitivity, but I dress myself according to my identity as a manager: grey colours.” 2. “These are places that I once visited and I was astonished by their beauty.”	1. Posing in different standing positions for the artist to make drawings of her 2. Formulating words that represent her inner experience of her identity	Colouring-in different drawings of the poses	Colouring-in a drawing of herself being posed as a confident woman, using very bright colours. Installing a picture of this portrait as profile picture on social media	“This full body portrait with passionate bright colours is a statement towards the world, where I am showing this self-expression.”
**9**	1. The story of the ‘Ant’s departure’ª is an inspiration to draw an ant. 2. Exploring art works in books by Morandi and Katharina Grosse	1. “The ant is caring and pays close attention to her surroundings. Her eyes radiate love- this is how I have been, but I am also afraid of people.” 2. Purple represents love, green represents nature and yellow represents divine trust	Drawing the theme ‘connection’ which is represented by two globes touching each other	An attempt to draw a pile of books, elements of a kitchen apron, several pots and pans and animals, including her dog, as she is practicing with drawing techniques to refine the pictures	Drawing the aforementioned elements again (books, pots and pans, kitchen apron, husband and dog)	“All important aspects of life are represented in drawings consisting of multiple elements. The connection to others is made visible.”
**10**	1. The story of the ‘The Metamorphosis’^a^ 2. Selecting pictures for a collage 3. Sensing fabrics 4. Plastering a mould of her hands	1. “The last sentence in the story touched me: ‘I could also ask for help’.” 2. The pictures remind her of her childhood 3. The fabrics bring up tenderness and resistance simultaneously 4. The hands represent giving and receiving	Creating a collage of pictures and fabrics that represent important elements in her life. Plastering another mould of her hands	Tearing apart all her diaries and making paper-mache out of the pieces	Creating a paper-mache bridge constructed of paper from shredded dairies, representing her spiritual transition from earthly life to the afterlife	“The bridge is being constructed out of my life stories. It is a cross over and meant for the transition to the afterlife, guided by those that are deeply connected to me.”

^a^In total 10 stories from world literature were selected and redacted for use within the ISOS project, see Kamp et al. 2021 [15] for details

Several patients started the co-creation process by building on the reading of selected literature (see 2.1), which constituted the start of phase 1.1 *Art communication, exploring through the senses*. Some other patients started the co-creation process from the content of the Rich Picture (see 2.1). The remaining patients started the co-creation process by visiting an arts museum together with the artist, by exploring the professional studio of the artist, by exploring photos from magazines, or sensing a set of objects arranged by the artist to initiate the co-creation process.

All patients explored various art forms during phase 1.1 *Art communication, exploring through the senses*, for example through drawings, literature, music and clay work. In phase 1.2 *Art communication, tracing aspects of the life narrative*, some patients explored multiple story lines within their life narrative while others explored one story line. During phase 2. *Element compilation*, inspired by phase 1, patients initiated the creation of a work of art while simultaneously reflecting upon and revaluating the experiences of contingency. The element compilation started rather broad (phase 2.1 *Element compilation, broad arrangement*) where patients tried out working with several art forms. Subsequently the use of art forms became more focussed towards the work of art (phase 2.2 *Element compilation, acuminating*). This process was finalized in phase 3. *Consolidation*, where the work of art was constructed. After finalizing the work of art, patients reflected upon both the work of art and on the process of arriving there in phase 4. *Reflection*. In all cases, patients followed the four phases of integration of experiences of contingency. In Table 4 these phases are presented with their characteristic content for each patient.

### Case description illustrating the phases of integration of experiences of contingency and the expression of experiences of contingency and ultimate life goals in the work of art

Here, we present a more detailed, yet compact, description, of a case which illustrates the process of integration of experiences of contingency and the expression of these experiences of contingency and ultimate life goals in the work of art.

This case describes a 45-year-old female patient who was currently undergoing chemotherapy for cervical cancer. The experience of contingency she addressed during the co-creation process was related to ‘loneliness’. She faced loneliness in the experience of having to manage the disease without support from her family, who lives abroad, and her partner, who was unable to sleep in the same room with her and was unavailable for emotional support due to his own grief of her having cancer. Also, grief of her inability of having children, as the problems with her uterus started in her adolescence, contributed to her experience of loneliness. Her worldview was strongly influenced by being catholic and originating from the Philippines. The ultimate life goal that was present during the co-creation was feeling love and sharing (God’s) love.

Prior to the co-creation process, together with a spiritual counsellor, she read a story called ‘Farewell from Phoebe’ written by Vonne van der Meer, which revolves around the theme of a woman losing her child. In her co-creation process, she explored images (1.1 *Art communications, exploring the senses*) that reminded her of her childhood with her parents. She also chose coloured wildflowers that reminded her of the myriad of feelings she was experiencing during the process of illness. The flowers slowly withered in time, which reminded her of the transience of her body and her inner fire being quenched. To her, white roses meant God’s guidance, and certain fabrics represented a dress that her mother had made for her when she was a child. (1.2 *Art communications, tracing aspects of the life narrative*). She was mostly drawn to creating something meaningful with wildflowers and white roses, and decided to make an arrangement of fresh flowers in the shape of a uterus, including her ovaries (2.1 *Element compilation, broad arrangement*). Pictures were made of the arrangement and then she decided to let the flowers wither and make new pictures of that same arrangement. The pictures were subsequently projected onto a white blanket to find the most fitting configuration, where the uterus’ shape was not clearly visible, but the colours of the flowers would stand out (2.2 *Element compilation, acuminating*). The configuration was printed onto the blanket. In the middle of one night, during a moment of despair, she wrote a poem addressing God where she asked for help to allow more love into her life. She wanted to complete the process by letting herself be photographed while being wrapped into the blanket (3. *Consolidation*). During the photo session she fell asleep in the blanket. The work of art, then, consisted of the blanket, a photograph of her sleeping in the blanket, and a recording of the poem she had written. She mentioned that the blanket represented several meanings: a warm wrapping of love, a place for shelter from the hardships of the illness, and a secret locker that stored her painful feelings regarding the lack of love she had experienced. Simultaneously, she felt that the blanket represented the new sense of love from her partner and family. She had found that she was in a phase to say: “let go and let God (i.e. ’I surrender to God’s plan’) – I will use the days that are left well, even if my time is going to be brief” (4. *Reflection*). During the co-creation process, she had started sharing her feelings with her partner and family thereby strengthening the connection between them.

**Fig. 1:**
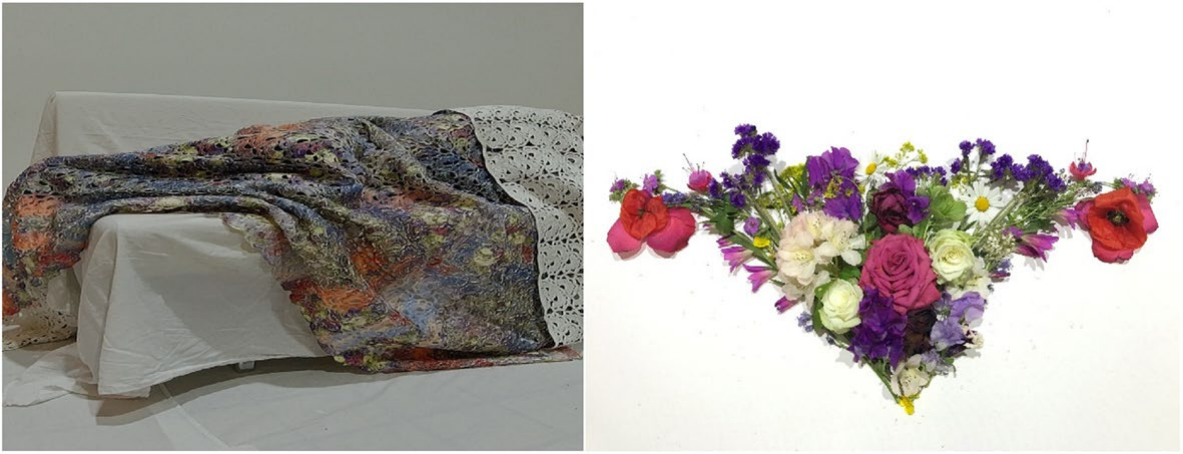
Case one, the patient sleeping in her blanket designed from printed flowers representing a uterus

In the works of art, her ultimate life goal was expressed in the blanket, in being wrapped in the blanket, and in the poem. This expression in the work of art was directly linked to the experience of contingency she had reflected upon: a sense of loneliness and a lack of support related to having cancer. The work of art entailed elements of love, connection and shelter. See Fig. 1 for an illustration.

## Discussion

The current study is the first explorative study in which the process of integration of experiences of contingency into one’s life narrative was observed as this unfolded in real time, in palliative cancer patients within a co-creation setting. The recordings of the co-creation sessions can be seen as the accounts of the patient’s developing life narrative, revealing what people value intrinsically [26].

In this study we found that both experiences of contingency and ultimate life goals were present within the co-creative process for all patients. The experiences of contingency they reflected upon during co-creation were linked to their particular ultimate life goal(s). These experiences of contingency and ultimate life goals were expressed in the work of art, indicating that they can be made explicit through a process of art making.

The current study illustrated how, during co-creation, all palliative cancer patients moved through the four phases of the process of integration of experiences of contingency into their life narrative. The current study showed that patients re-appraise their experiences of contingency during co-creation. Yet, the full effect of this integration of experiences of contingency on the coherence and meaning of a patient’s life narrative remains to be further investigated.

Although further research is needed, the current study offered some indication that dealing with an experience of contingency was intimately related to patients’ ultimate life goals. Palliative patients, whether conscious or not, were found to increasingly try to visualize the fulfilment of these ultimate life goals within the co-creation process. The model of Hartog et al. [2] hypothesizes that people try to fulfil their ultimate life goals. When confronted with the finitude of one’s life, as in being in a palliative state, the fulfilment of these ultimate life goals could become more urgent, prominent or inevitable.

In the case of irreversible changes that are impeding the achievement of one’s life goals, it could be beneficial to shift attention to goals that are still attainable [27]. The main themes of ultimate life goals present in our study, intimacy/relationships, self-transcendence/generativity and spirituality/religiosity, might be more attainable for palliative cancer patients than ultimate life goals regarding work/achievement, a theme which was not present in our study. We do not know whether patients focus on specific existing ultimate life goals or develop new ultimate life goals during the trajectory of their palliative illness. It could be hypothesised that focus on attainable life goals supports patients to endure the experience of contingency.

The current study illustrates how palliative cancer patients can move through the four phases of integration of experiences of contingency into their life narrative within a co-creation setting as theorized previously by our group [16]. The work of art could be described as a solidification of contingency [28], creating an opportunity to perceive their experiences from a distance through the work of art. When patients interact with the work of art they can both recognise themselves again and recognise themselves anew [29]. This could include self-transcendence when their sense of self becomes changed [2, 3, 10]. The experience of contingency arising from a rupture of one’s life narrative could provide a possibility for the start of a new chapter of this very life narrative. Palliative cancer patients using their creativity in co-creation, seem to look for and find new ways to continue to fulfil their ultimate life goals, regardless of the changing circumstances. This might support the (discovery of) sustainment of core aspects of their identity and importance or meaning of their life [30].

An interesting question is to what extend the various elements of the co-creation process contributed to our findings. Earlier research indicated that attunement between artist and patient supports the integration of experiences of contingency [16, 21]. An important factor for creative processes aiming at self-reflection is that the patient needs to feel safe in a setting which contributes to imagination and creativity, and provides possibilities for expression in art modalities. The choice of the locations for the co-creation processes were adapted to fit these needs [31]. Due to decreased mobility in some patients ISOS offered the possibility for co-creation sessions to be held at the patients home. The artists studio contributed to imagination and stimulated creativity in patients, as did visiting a museum or trips into nature. Yet, we do not know how the various settings have affected the creation of the work of art and the integration of experiences of contingency into the life narrative, which could be a subject of future research. A limitation of the study and for that matter, the co-creation process, is that it only included patients who were motivated to be confronted with their illness and were able to participate. The patients of the current study who finalised the work of art, attended all scheduled sessions and provided input for those sessions, which may, in part, explain the coherent unfolding of the co-creation processes. At the end of the co-creation process, all patients reported to embrace life (again). Another limitation of the study is that the natural unfolding of the co-creation processes needed more time than expected, possibly contributing to attrition as at the moment of analysis four patients had dropped out due to deteriorating health. A methodological limitation is that the data analysis was not performed independently by several researchers which does not allow for interrater comparison and establishment of interrater reliability. A strength of the study is the participation of professional artists who were skilled in their profession, able to co-create a work of art, and trained to listen and attune to the input of patients enabling the co-creation process and the integration of the experiences of contingency patients reflected upon. The studied population is rather narrow and this limits the generalisability of the findings. In a palliative setting people are confronted with end of life issues, possibly enhancing motivation in some patients to deal with these issues and integrate such experiences of contingency into their life narrative. Yet, it is a universal given that people are confronted with negative life events of all sorts giving rise to experiences of contingency. As such, the intervention might also be supportive for the integration of other adverse life events than the experience of advanced cancer, for instance, in other palliative settings and chronic illnesses.

## Conclusions

During co-creation, experiences of contingency and ultimate life goals, made explicit through a process of art making, are expressed in the work of art. These experiences of contingency become integrated into the life narrative by moving through four phases; *Art communications, Element compilation, Consolidation, Reflection.*

Interventions aiming to support patients dealing with experiences of contingency through a process of art making, while focussing on life goals, and reflecting on experiences of contingency with a professional artist, have the potential to assist the integration of experiences of contingency into one’s life narrative.

## Data Availability

Audio recordings are available upon request. Requests can be send to Yvonne Weeseman at y.weeseman@amsterdamumc.nl.
